# Inoculation with *Stutzerimonas stutzeri* strains decreases N₂O emissions from vegetable soil by altering microbial community composition and diversity

**DOI:** 10.1128/spectrum.00186-24

**Published:** 2024-03-21

**Authors:** Nan Gao, Huanhuan Zhang, Chun Hu, Qing Li, Linmei Li, Peng Lei, Hong Xu, Weishou Shen

**Affiliations:** 1 Department of Biological Engineering, School of Biotechnology and Pharmaceutical Engineering, Nanjing Tech University, Nanjing, China; 2 Jiangsu Key Laboratory of Atmospheric Environment Monitoring and Pollution Control, Collaborative Innovation Center of Atmospheric Environment and Equipment Technology, and School of Environmental Science and Engineering, Nanjing University of Information Science and Technology, Nanjing, China; 3 School of Food Science and Light Industry, Nanjing Tech University, Nanjing, China; Oulun yliopisto, Oulu, Finland

**Keywords:** microbial community composition, nitrogen-cycle functional genes, nitrous oxide, plant growth-promoting rhizobacteria (PGPR), soil texture

## Abstract

Inoculation with plant growth-promoting rhizobacteria (PGPR) strains has promoted plant growth and decreased nitrous oxide (N₂O) emissions from agricultural soils simultaneously. However, limited PGPR strains can mitigate N₂O emissions from agricultural soils, and the microbial ecological mechanisms underlying N₂O mitigation after inoculation are poorly understood. In greenhouse pot experiments, the effects of inoculation with *Stutzerimonas stutzeri* NRCB010 and NRCB025 on tomato growth and N₂O emissions were investigated in two vegetable agricultural soils with contrasting textures. Inoculation with NRCB010 and NRCB025 significantly promoted tomato growth in both soils. Moreover, inoculation with NRCB010 decreased the N₂O emissions from the fine- and coarse-textured soils by 38.7% and 52.2%, respectively, and inoculation with NRCB025 decreased the N₂O emissions from the coarse-textured soil by 76.6%. Inoculation with NRCB010 and NRCB025 decreased N₂O emissions mainly by altering soil microbial community composition and the abundance of nitrogen-cycle functional genes. The N₂O-mitigating effect might be partially explained by a decrease in the (*amoA + amoB*)/(*nosZ*I + *nosZ*II) and (*nirS + nirK*)/(*nosZ*I + *nosZ*II) ratios, respectively. Soil pH and organic matter were key variables that explain the variation in abundance of N-cycle functional genes and subsequent N₂O emission. Moreover, the N₂O-mitigating effect varied depending on soil textures and individual strain after inoculation. This study provides insights into developing biofertilizers with plant growth-promoting and N₂O-mitigating effects.

**IMPORTANCE:**

Plant growth-promoting rhizobacteria (PGPR) have been applied to mitigate nitrous oxide (N₂O) emissions from agricultural soils, but the microbial ecological mechanisms underlying N₂O mitigation are poorly understood. That is why only limited PGPR strains can mitigate N₂O emissions from agricultural soils. Therefore, it is of substantial significance to reveal soil ecological mechanisms of PGPR strains to achieve efficient and reliable N₂O-mitigating effect after inoculation. Inoculation with *Stutzerimonas stutzeri* strains decreased N₂O emissions from two soils with contrasting textures probably by altering soil microbial community composition and gene abundance involved in nitrification and denitrification. Our findings provide detailed insight into soil ecological mechanisms of PGPR strains to mitigate N₂O emissions from vegetable agricultural soils.

## INTRODUCTION

Nitrous oxide (N₂O), an important greenhouse gas that remains in the atmosphere for a long time and destroys the ozone layer, has a global warming potential 298 times that of carbon dioxide over 100 years, contributing to 5%–6% of the heat trapped by all greenhouse gases (1, 2). Agricultural sources are the major source of anthropogenic N₂O emissions globally (3–5). Microbial N₂O production pathways in soil are relatively complex and diverse, including nitrification, denitrification, nitrogen (N)-dissimilated reduction to ammonium, denitrification by nitrifying microorganisms, and comammox (6). Nitrification and denitrification are the main pathways of N₂O production in agricultural soil (5, 7, 8). Nitrification occurs when microorganisms oxidize ammonia (NH_3_) to nitrite (NO_2_^−^) or nitrate (NO_3_^−^) with N₂O as a byproduct in an aerobic environment. Among them, NH_3_ oxidation encoded by *amoA* is a crucial step in producing the N_2_O (4). Denitrification is a process in which soil microorganisms reduce NO_2_^−^ or NO_3_^−^ to gaseous NO, N₂O, or N₂ under the catalysis of a series of enzymes in an anoxic environment (4, 8, 9). Among denitrification steps, NO_2_^−^ reductases (encoded by *nirK* or *nirS*), which catalyze NO_2_^−^ reduction to NO, and N₂O reductases (encoded by *nosZ*), which catalyze N_2_O reduction to N₂, are considered the most important sources and sinks of soil N₂O (10).

Nitrification and denitrification are affected by soil physicochemical properties and bacterial community composition (1, 9, 11). Soil physicochemical properties, such as soil texture, organic matter (SOM), available N, and pH, are critical factors affecting N₂O emissions (12, 13). Fine-textured soils may release less N₂O than coarse-textured soils, which restrains gas diffusivity and enhances N₂O reduction to N₂ through denitrification (5, 11, 14, 15). SOM and available N provide carbon (C), N, and energy sources for microbial nitrification and denitrification and are, therefore, dominant factors regulating N₂O emissions (2, 11, 16). In microcosm experiments, straw amendment altered denitrifying microbial community composition by increasing SOM content in silty and clay soil, decreasing N₂O emissions (11). The application of *Trichoderma viride* EBL13 biofertilizer increased soil-dissolved organic C, which can be used as a C source by heterotrophic denitrifiers to enhance the reduction of N₂O to N₂ (17). Application of a bio-organic fertilizer containing *Trichoderma guizhouense* NJAU 4742 resulted in N_2_O emissions being significantly less than those of synthetic fertilizers, possibly because the NH_4_^+^-N content decreased and the nitrification was abated (2). Soil pH is a dominant factor that determines functional microbial communities in the N cycle, especially the growth, activity, and community composition of archaeal and bacterial *amoA* and *amoB* that encodes ammonia monooxygenase, influencing N₂O emissions from soils (18). The ratio of N₂O to N₂ decreases with increasing pH. For example, inoculation with *Bacillus amyloliquefaciens* EBL11 increased soil pH, decreased archaeal and bacterial *amoA* gene abundance, and decreased N₂O emissions from acidic soils (1).

Exogenous additives affect the soil microbial community and N-cycle gene abundance associated with N₂O production and consumption (2, 17, 19). Inoculation with *T. viridis* EBL13 significantly increased the diversity of the N₂O-reducing community and stimulated the expression of *nirK*, *nirS*, and *nosZ*, decreasing soil N₂O emissions (17). Bio-organic fertilizer containing *T. guizhouense* NJAU 4742 reduced bacterial *amoA* abundance, increased *nosZ* abundance, and decreased N_2_O emissions from soils (2). Straw amendment decreased the abundance of denitrifying Proteobacteria and increased the abundance of N₂O-reducing Clostridium in clay soil, decreasing N₂O emissions from soils (13). However, the straw amendment did not affect the N₂O emissions from silty soil (13). According to the literature, the only known N₂O sink is the biochemical reduction of N₂O to N_2_ by N₂O-reducing microorganisms that possess the *nosZ* gene (3, 7). Furthermore, due to different *nosZ* expression levels or N₂O reductase activities, bacteria possessing *nosZ* genes may decrease different percentages of N₂O in soils (4, 20).

Several mitigation technologies and practices have been attempted to reduce N₂O emissions from agricultural soil, mainly focusing on the effects of N fertilizer type, application rates, biochar applications, and conservation tillage on N₂O emissions (2, 10, 11, 16, 19). Moreover, the application of plant growth-promoting rhizobacteria (PGPR) with N₂O mitigation abilities has become a promising method (1, 5, 20) owing to its simultaneous N₂O mitigation and crop growth promotion. PGPR mitigates N₂O emissions based on the strain, soil physical and chemical properties, and crop species. Inoculation with *Azospirillum sp*. TSA2s, *Azospirillum sp*. TSH100, *Herbaspirillum sp*. UKPF19, and *Herbaspirillum sp*. UKPF54 changed soil C and N contents and significantly decreased cumulative N₂O emissions from two Fluvisol soils planted with red clover and timothy (5). Inoculation with *B. amyloliquefaciens* EBL11 changed the soil pH and mineral N contents, decreased bacterial *amoA* gene abundance, and increased N₂O-reducing bacteria abundance, decreasing N₂O emissions from an acidic soil planted with oil-seed rape in a greenhouse pot experiment (1). Inoculation with *Azospirillum sp*. UNPF1 significantly decreased cumulative N₂O emissions in red clover soil but did not in timothy soil (5). Thus, only certain PGPR strains can mitigate N_2_O emissions from soils, and the effects of PGPR on N_2_O emissions may be related to soil texture, strain effect, and crop species. Moreover, the ecological mechanisms underlying soil microbial communities to N₂O mitigation are poorly understood after inoculation with these effective strains.

Two strains, *Stutzerimonas stutzeri* (previously classified as *Pseudomonas stutzeri*) NRCB010 and NRCB025, have shown promising plant growth-promoting effects on agar plate, possibly through producing auxin, siderophore, and solubilizing phosphate. Additionally, they showed NO_3_^–^-N removal abilities from agricultural wastewater through denitrification (21). Thus, to improve the understanding of the N₂O mitigation ability of NRCB010 and NRCB025 in soil, this study conducted greenhouse pot experiments in two differently textured vegetable agricultural soils. The aims were to (i) investigate the effects of inoculation with NRCB010 and NRCB025 on N₂O emissions and tomato growth in two vegetable agricultural soils and (ii) explore the underlying mechanisms of decreases in N₂O emissions after the inoculation with these two PGPR strains.

## RESULTS

### Effects of *S. stutzeri* on tomato growth, soil physicochemical properties, and N₂O emissions from two soils

**Fig 1:**
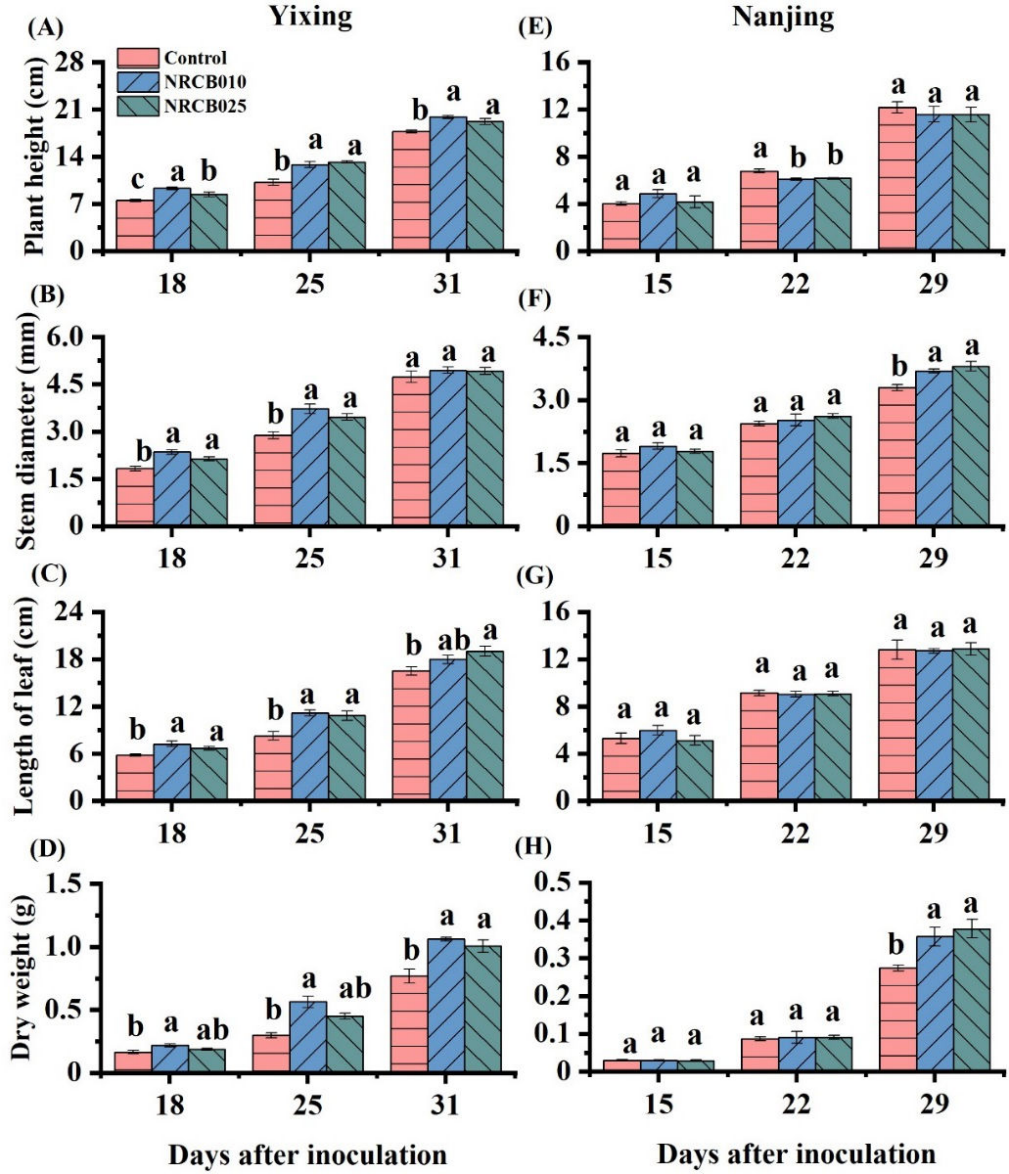
Plant height (**A and E**), stem diameter (**B and F**), length of leaf (**C and G**), and dry weight (**D and H**) of tomato after inoculation with *S. stutzeri* strains in Yixing (A, B, C, and D) and Nanjing (E F, G, and H) soil. Values are mean ± SE (*n* = 4). Letters above the bars at the same time denote significant differences between treatments of the same soil revealed by Duncan’s post hoc test (*P* < 0.05).

This study examined two soils: Yixing soil and Nanjing soil. Inoculation with *S. stutzeri* significantly improved tomato growth. Plant height significantly increased at 31 days after inoculation (DAI) for both strains in the Yixing soil (Fig. 1A), and stem diameter significantly improved at 29 DAI in both strains in the Nanjing soil (Fig. 1F). At harvest, the dry weights were significantly higher after inoculation with NRCB010 and NRCB025 than those of the control, increasing by 38.1% and 30.7% in the Yixing soil (Fig. 1D) and by 30.3% and 37.9% in the Nanjing soil (Fig. 1H), respectively.

Inoculation with *S. stutzeri* strains significantly affected soil pH, SOM, and NH_4_^+^-N contents (Table 1; Table S1). In the Yixing soil, NRCB010 significantly increased the pH at 18 and 31 DAI, and NRCB025 significantly decreased the pH at 18 and 25 DAI. In the Nanjing soil, NRCB010 significantly decreased the pH at 15 DAI, and NRCB025 significantly decreased the pH at 21 and 29 DAI. NRCB010 and NRCB025 significantly decreased the SOM content at 25 DAI in the Yixing soil and increased the SOM content at 22 DAI in the Nanjing soil. NRCB010 and NRCB025 significantly increased the NH_4_^+^-N content at 22 DAI in the Nanjing soil (Table S1).

**TABLE 1:** Physicochemical properties of tomato soil after inoculation with *S. stutzeri* strains^*a*^

Treatment	pH (H_2_O)			SOM (g kg^−1^)		
Yixing soil						
DAI	18	25	31	18	25	31
Control	5.28 ± 0.07b	5.47 ± 0.06a	5.00 ± 0.03c	12.8 ± 0.5a	15.3 ± 0.4a	15.1 ± 0.2a
NRCB010	5.52 ± 0.03a	5.01 ± 0.03c	5.76 ± 0.09a	14.0 ± 0.1a	12.7 ± 0.9b	15.7 ± 0.9a
NRCB025	5.12 ± 0.02c	5.32 ± 0.03b	5.47 ± 0.02b	13.9 ± 1.2a	13.1 ± 0.4b	14.6 ± 0.4a
Nanjing soil						
DAI	15	22	29	15	22	29
Control	5.67 ± 0.08c	5.92 ± 0.04a	5.61 ± 0.02a	10.1 ± 0.3a	8.5 ± 0.2b	10.9 ± 1.0b
NRCB010	5.86 ± 1.05b	5.77 ± 0.07ab	5.66 ± 0.04a	7.9 ± 0.3b	10.3 ± 0.6a	11.2 ± 0.2b
NRCB025	6.09 ± 0.02a	5.66 ± 0.03b	5.38 ± 0.04b	8.5 ± 0.2b	10.3 ± 0.2a	13.4 ± 0.4a

^*a*^ Values are mean ± SE (*n* = 4). Letters after the data in each volume denote significant differences between different treatments revealed by Duncan's post hoc test (*P* < 0.05).

**Fig 2:**
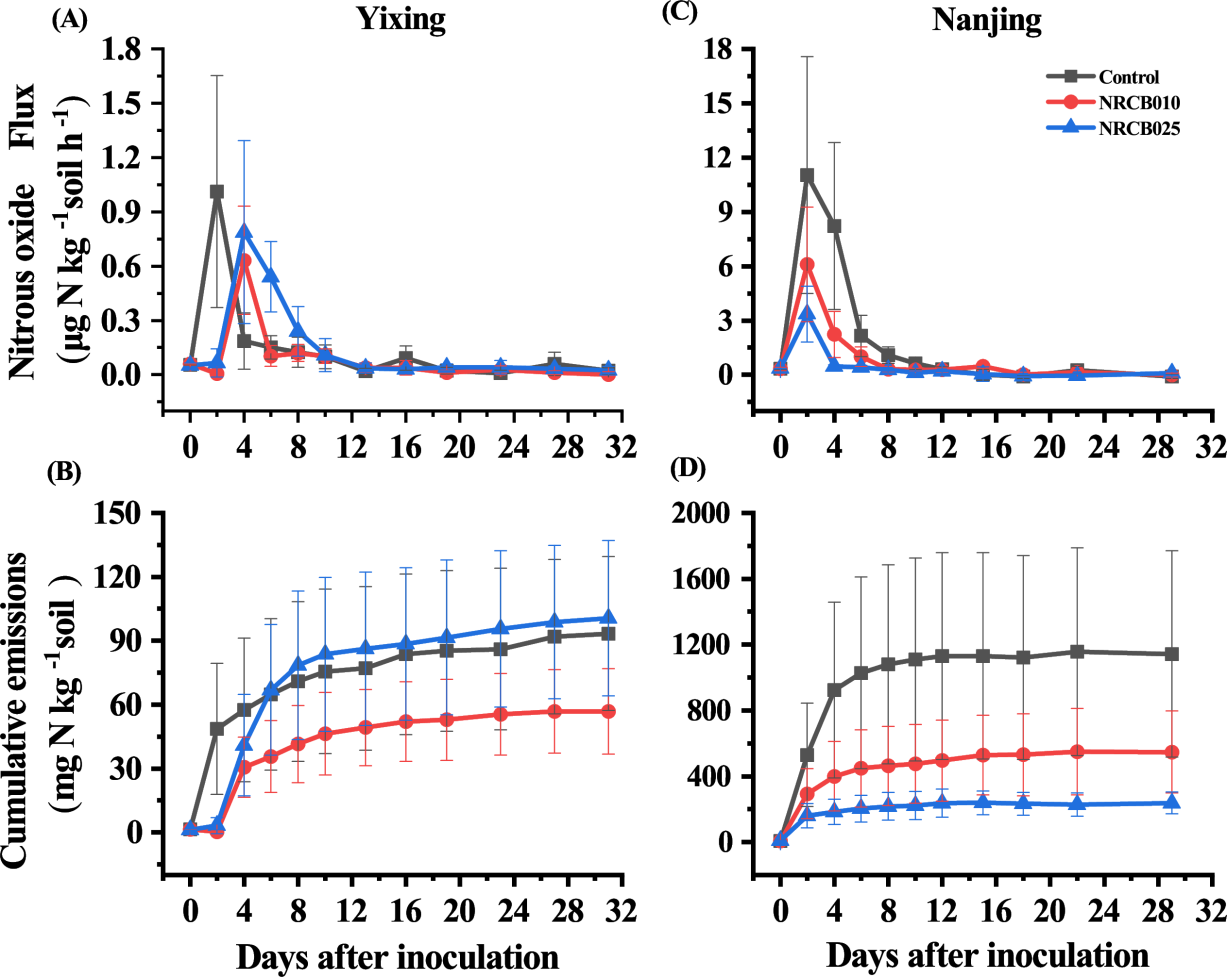
N_2_O flux (**A and C**) and cumulative emission (**B and D**) from Yixing (**A and B**) and Nanjing (**C and D**) soil after inoculation with *S. stutzeri* strains. Values are mean ± SE (*n* = 4).

A two-factor analysis of variance (ANOVA) indicated that inoculation with *S. stutzeri* significantly affected N₂O emissions depending on the strain and soil source (Table 2). In the Yixing soil, N₂O flux was lower at 2 DAI in response to NRCB010 and NRCB025 and higher at 4 DAI when compared with that of the control (Fig. 2A). NRCB010 decreased cumulative N₂O emissions, which were 38.7% less at 31 DAI than those of the control (Fig. 2B). Inoculation with NRCB025 did not decrease cumulative N₂O emissions from 6 DAI onward (Fig. 2B). In the Nanjing soil, N₂O flux was lower at 2 and 4 DAI in response to NRCB010 and NRCB025, respectively, than that of the control (Fig. 2C). Inoculation with NRCB010 and NRCB025 decreased the N₂O cumulative emissions, which were 52.2% and 76.6% less at 31 DAI than those of control, respectively (Fig. 2D).

**TABLE 2:** Variance analysis of PGPR effect and soil *t* source on N_2_O emissions and soil physicochemical properties in all sample treatments^*a*^

Items	N_2_O emissions		pH		SOM	
	F	*P*	F	*P*	F	*P*
PGPR effect (*P*)	5.069	**<0.001**	1.436	0.245	0.207	0.813
Soil source (S)	22.281	**<0.001**	53.061	**<0.001**	100.162	**<0.001**
*P* × S	5.054	**<0.001**	0.587	0.559	1.199	0.308

^*a*^ PGPR effect: control, NRCB010, and NRCB025. Soil source: Yixing soil and Nanjing soil. Results were obtained using two-way ANOVA with PGPR effect and soil texture as fixed effects. *P* value < 0.05 was indicated in bold.

### Effects of *S. stutzeri* on bacterial diversity in tomato rhizospheric soil

**Fig 3:**
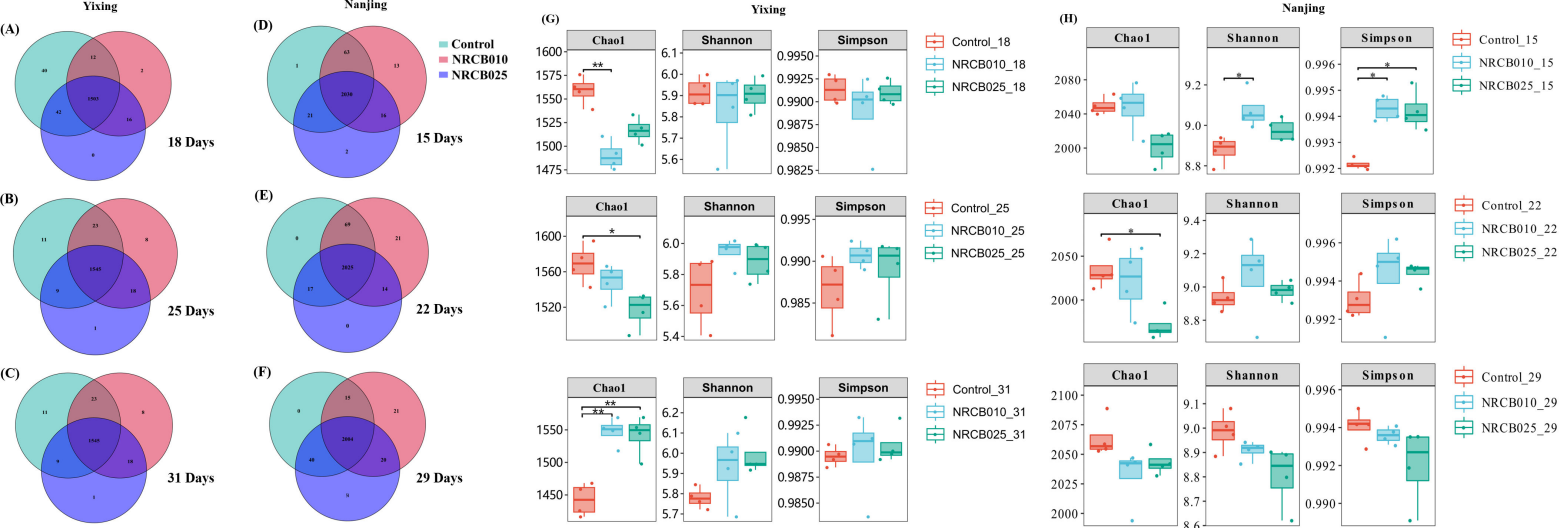
Bacterial diversity after inoculation with *S. stutzeri* strains in Yixing (A, B, C, and G) and Nanjing (D, E, F, and H) soils. (**A–F**) Venn diagram of similarity and overlap of microbial composition. (**G–H**) Microbial alpha diversity index. Differences in microbial alpha diversity index were assessed between treatments; the significance levels are denoted by * for *P* < 0.05 and ** for *P* < 0.01.

Inoculation with *S. stutzeri* strains affected operational taxonomic units (OTUs) and unique OTU numbers, depending on the strain and soil source. A total of 3,746 OTUs were obtained from all 72 soil samples: 1,615 OTUs from the Yixing soils with 27 phyla and 349 genera in species annotation and 2,146 OTUs from the Nanjing soils with 27 phyla and 253 genera species annotation. A high percentage of OTUs was shared between each treatment in the same soil (Fig. 3A through F). In the Yixing soil, the unique OTU numbers of the control were higher than those of the NRCB010 and NRCB025 treatments (Fig. 3A through C). In the Nanjing soil, the unique OTU numbers of the control were lower than those of NRCB010 and NRCB025 treatments at 15 and 29 DAI (Fig. 3E and F).

Inoculation with *S. stutzeri* strains affected alpha diversity depending on the strain and soil source. In the Yixing soil, Chao1 indices of the control were significantly lower than those of NRCB010 inoculated at 18 and 31 DAI and were significantly lower than those of NRCB025 inoculated at 25 and 31 DAI (Fig. 3G). The Shannon and Simpson indices were not significantly different among treatments. In the Nanjing soil, the Shannon and Simpson indices significantly increased after 15 d when inoculated with NRCB010; the Chao1 index significantly decreased after 22 DAI when inoculated with NRCB025 (Fig. 3H).

Principal coordinate analysis based on the Bray-Curtis distance revealed the influence of soil source and strain inoculation on soil bacterial communities (Fig. S1). In the Yixing soil, at 18 and 31 DAI, the bacterial communities in the NRCB010 and NRCB025 treatments overlapped but exhibited more diverse communities than those of the control (Fig. S1A). In the Nanjing soil, the NRCB010, NRCB025, and control groups were separated at 18 and 22 DAI (Fig. S1B).

### Effects of inoculation with *S. stutzeri* on microbial community composition and predicted functions in tomato rhizospheric soil

**Fig 4:**
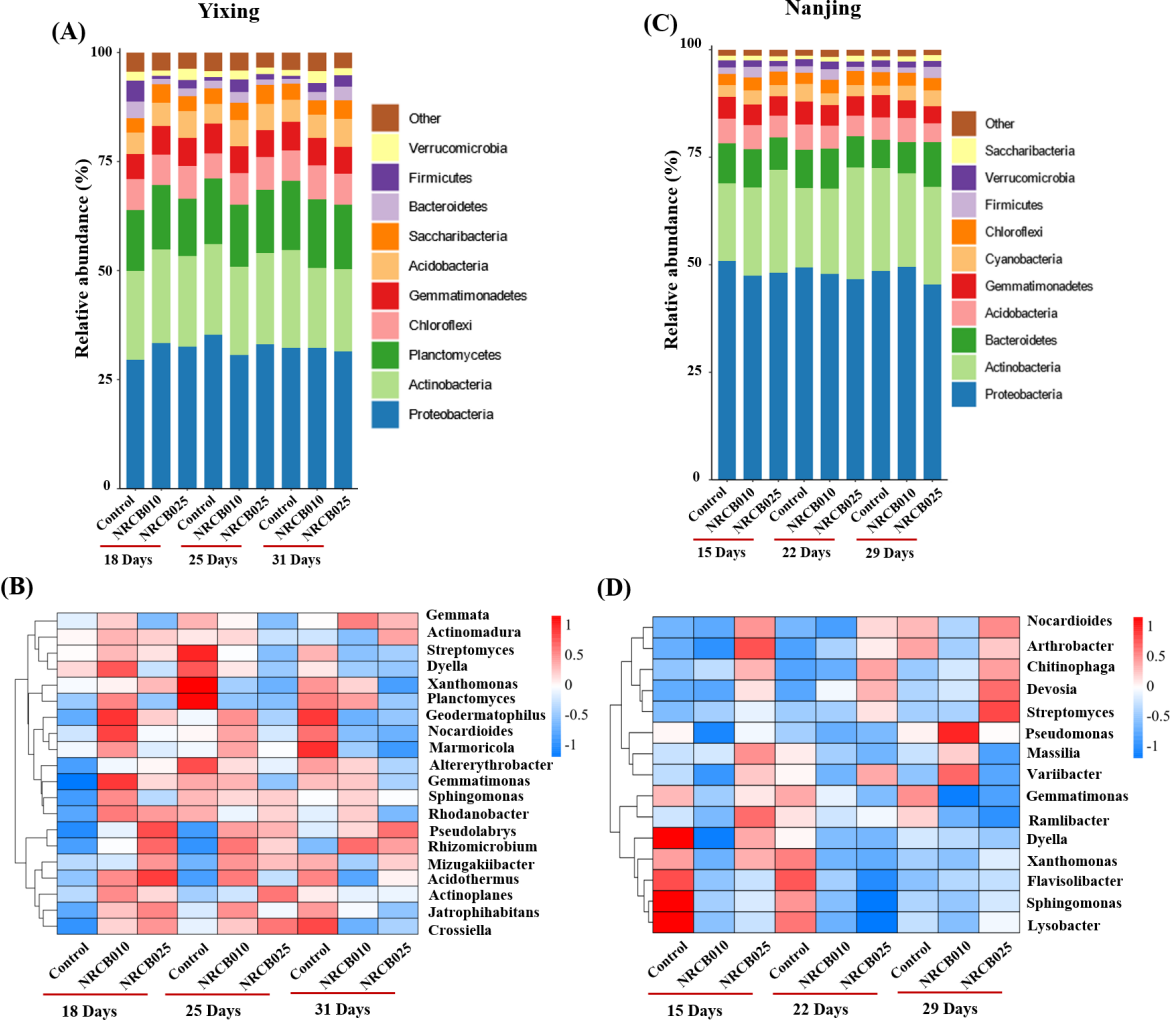
Rhizospheric microbial composition of tomato under different treatments in Yixing and Nanjing soils. (**A and C**) Bar diagram of root microbial composition of tomato root under different treatments at the phylum level. (**B and D**) Heat maps of similarities and differences in the community composition of genus level under different treatments.

For the same soil, the microbial populations of all treatments were similar at the phylum level. However, they differed in relative abundances (Fig. 4). In the Yixing soil, the three most abundant phyla were Proteobacteria, Actinobacteria, and Planctomycetes (Fig. 4A). At 18 DAI, inoculation with NRCB010 increased the relative abundances of the top three phyla, which were 3.9%, 1.1%, and 0.9% higher than those of control, respectively. Inoculation with NRCB025 increased the relative abundance of Proteobacteria by 3.1%. In the Nanjing soil, the three most abundant phyla in all treatments were Proteobacteria, Actinobacteria, and Bacteroidetes (Fig. 4C). The relative abundance of Actinobacteria was 1.4%–7.5% at 15 and 22 DAI higher than that of the control, and the relative abundance of Bacteroidetes was 0.8%–3.9% at 29 DAI after inoculation with NRCB010 and NRCB025 higher than that of the control (Fig. 4C). At 29 DAI, inoculation with NRCB010 increased the relative abundance of Proteobacteria, which were 1.0% higher than that of the control (Fig. 4C).

The composition of the bacterial communities in these two soils at the genus level is shown in Fig. 4B and D. In the Yixing soil, the three most abundant genera in all samples were *Sphingomonas*, *Gemmatimonas*, and *Rhodanobacter* (Fig. 4B). At 18 DAI, the relative abundances of the three genera in the NRCB010 group were 1.4%, 2.1%, and 1.4% higher, respectively, than those of the control (Fig. 4B). Furthermore, inoculation with NRCB025 increased the relative abundances of *Gemmatimonas* and *Rhodanobacter* by 1.4% and 1.2%, respectively, at 18 DAI (Fig. 4B). Similarly, in the Nanjing soil, the top three most abundant genera among the 15 genera were *Sphingomonas*, *Pseudomonas*, and *Gemmatimonas* (Fig. 4D).

**Fig 5:**
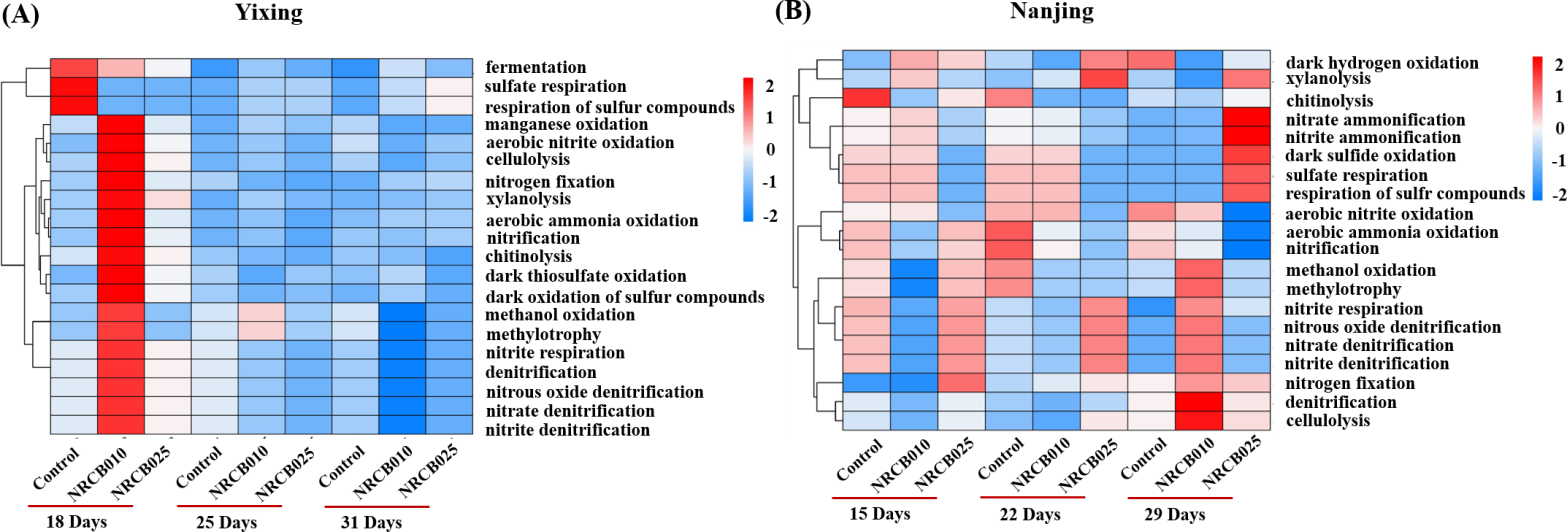
Heatmap of functional microbial community profiles of different treatments in Yixing (**A**) and Nanjing (**B**) soils based on FAPROTAX.

The predicted bacterial functional analysis showed that the gene abundances involved in NO_3_^−^ denitrification, NO_2_^−^ denitrification, N₂O denitrification, and NO_2_^−^ respiration increased by 7.9%–62.6% at 18 and 29 DAI after inoculation with NRCB010 in the Yixing and Nanjing soils, respectively (Fig. 5). The abundance of these genes in the Nanjing soil increased by 27.1%–36.6% at 22 DAI after inoculation with NRCB025 (Fig. 5B). Nitrification gene abundance increased by 134.2% 18 DAI after inoculation with NRCB010 in the Yixing soil, and no significant changes were observed when inoculated with NRCB025 (Fig. 5A).

### Effects of inoculation with *S. stutzeri* on N-cycle functional genes in tomato rhizospheric soil

**Fig 6:**
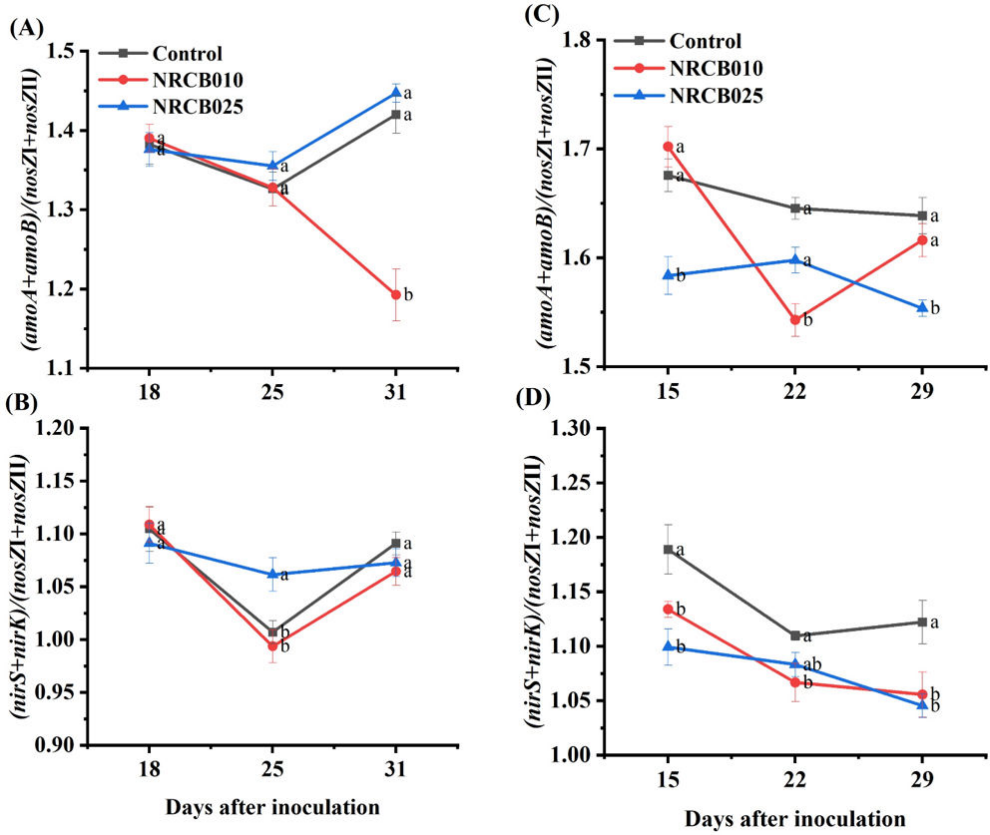
Dynamics of the copy number ratio of (*amoA + anoB*)/(*nosZ*I + *nosZ*II) and (*nirS + nirK*)/(*nosZ*I + *nosZ*II) under different treatments in Yixing (**A and B**) and Nanjing (**C and D**) soils. Values are mean ± SE (*n* = 4). Letters above the bars at the same time denote significant differences between treatments of the same soil revealed by Duncan’s post hoc test (*P* < 0.05).

Nitrification- and denitrification-related gene copy numbers and the ratio of key gene copy numbers for N₂O production to reduction changed depending on the strain, soil source, and inoculation duration (Fig. 6; Fig. S2 and S3). In the Yixing soil, the ratio of (*amoA + amoB*)/(*nosZ*I + *nosZ*II) was significantly lower at 31 DAI after inoculation with NRCB010 than that in the control (Fig. 6A). Moreover, the ratio of (*nirK + nirS*)/(*nosZ*I + *nosZ*II) was significantly higher at 25 DAI after inoculation with NRCB025 than that in the control (Fig. 6B). In the Nanjing soil, the ratio of (*amoA* + amoB)/(*nosZ*I + *nosZ*II) significantly decreased 22 DAI after inoculation with NRCB010, and it significantly decreased at 15 and 29 DAI after inoculation with NRCB025 (Fig. 6C). The ratio of (*nirK + nirS*)/(*nosZ*I + *nosZ*II) was significantly lower at 15 and 29 DAI after inoculation with NRCB010 and NRCB025, respectively, than that in the control (Fig. 6D).

### Drivers of N₂O cumulative emissions in tomato rhizospheric soil

**Fig 7:**
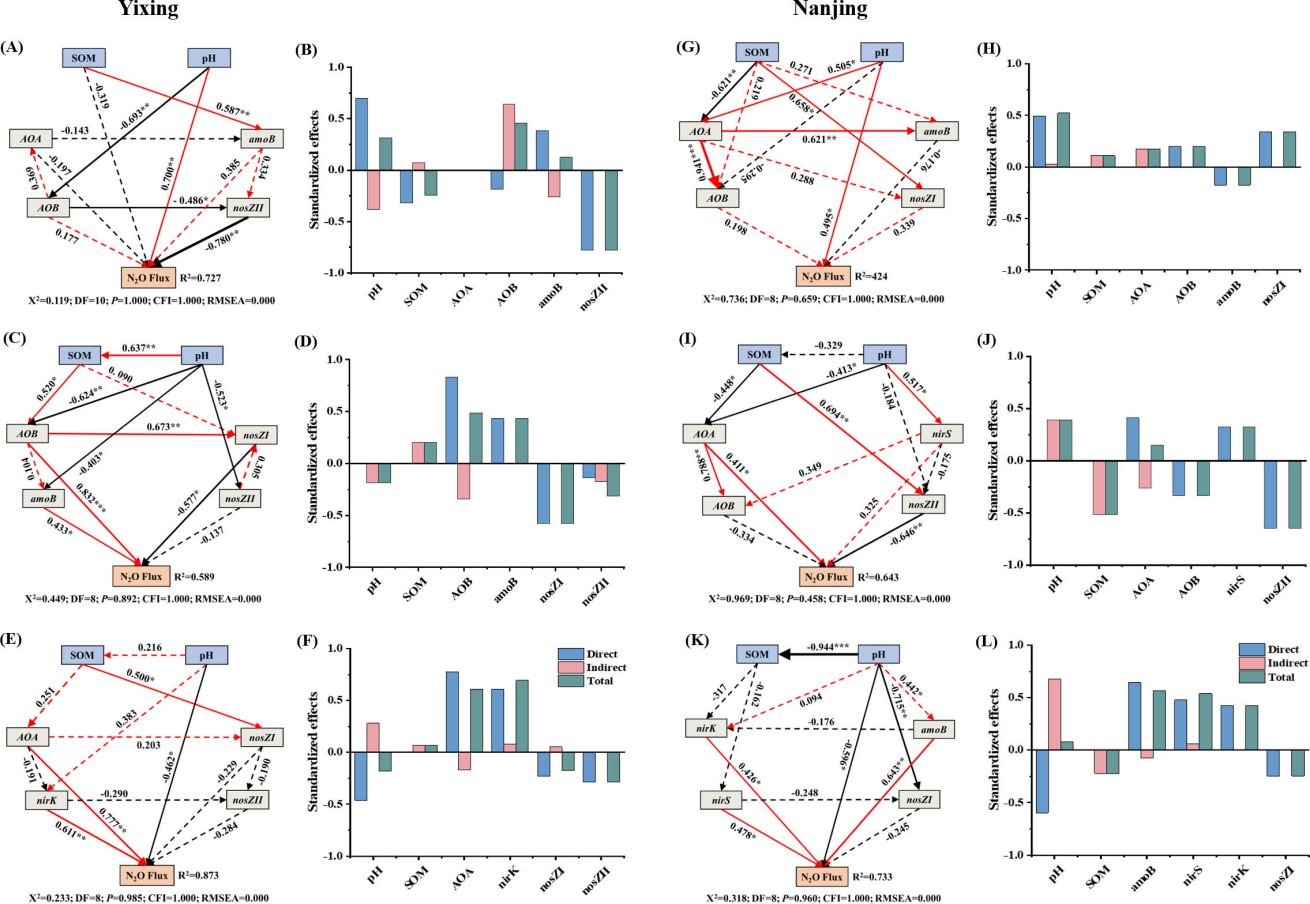
SEM (A: Control; C: NRCB010; E: NRCB025; in the Yixing soil; G: Control; I: NRCB010; K: NRCB025; in the Nanjing soil) showing relationships between soil physicochemical properties, N-cycle functional gene copy numbers, and N_2_O emission flux among different treatments. Standardized total effect (±bootstrap 95% CI; B: Control; D: NRCB010; F: NRCB025; in Yixing soil; H: Control; J: NRCB010; L: NRCB025; in Nanjing soil) based on SEMs. The red solid line represents the positive effect, the black solid line represents the negative effect, and the dashed line represents the nonsignificant path. Line thickness indicates the strength of the standardized path coefficients. Numbers on the line indicate significant standardized path coefficients (*P < 0. 05; **P < 0.01; ***P < 0.001) proportional to the line width. *R*^2^ indicates the variance of endogenous variables explained by the model. Goodness-of-fit statistics: χ^2^, CMIN/DF; DF, degrees of freedom; *P*, probability level; CFI, comparative fit index; RMSEM, root mean square error of approximation.

In addition to soil source being significantly correlated with N₂O emissions (Table 2), soil physicochemical properties (e.g., pH and SOM) were directly and indirectly correlated with N-cycle functional genes (Fig. 7). Structural equation modeling (SEM) showed that the soil physicochemical properties, nitrification, and denitrification of the total variation in N₂O emissions accounted for 72.7%, 58.9%, and 87.3% in the Yixing soil and 42.4%, 64.3%, and 74.5% in the Nanjing soil, respectively (Fig. 7). In both soils, in the control group, pH had a significantly positive and direct effect on N₂O emissions. Moreover, in the NRCB010 group, archaeal and bacterial *amoA* gene copy numbers had a significantly positive and direct effect, and *nosZ* (I or II) gene copy numbers had a significantly negative and direct effect on N₂O emission. In the NRCB025 group, *nirK* gene copy numbers had a significantly positive and direct effect, and pH had a significantly negative and direct effect on N₂O emission. In addition, soil physicochemical properties and partial N-cycle functional genes had multiple indirect effects on N₂O flux (Fig. 7). The standardized total effect of SEM showed that the gene copy numbers of *nosZ*II, *nosZ*I, and *nirK* were the greatest direct factors affecting N₂O emissions from the Yixing soil, respectively; soil pH, *nosZ*II, and *amoB* were the greatest direct factors affecting N_2_O emissions from the Nanjing soil, respectively (Fig. 7).

## DISCUSSION

### Effects of PGPR on crop growth and soil N₂O emissions

PGPR, an exogenous bacterium, when inoculated into the soil, sometimes does not survive or does not have the same performance in different soil conditions because they must compete with better-adapted native microorganisms (22). Therefore, identifying specific strains is important and would enhance crop growth, mitigate soil N₂O emission, and facilitate adaptation to a specific soil condition. Some reports have evaluated *S. stutzeri* or *Pseudomonas sp*. evaluated based on soil conditions. Examples of their results are as follows: inoculation with *P. aeruginosa* RP2 increased groundnut dry weight in a greenhouse pot experiment (23); *P. stutzeri* A1501 promoted the growth of maize in greenhouse pots and in field experiments (24); and inoculation with NRCB010 and NRCB025 promoted tomato seedling growth on agar plates (21). Similar to these results, in this study, inoculation with NRCB010 and NRCB025 significantly increased the dry weight of tomatoes in a greenhouse pot experiment using two vegetable agricultural soils (Fig. 1). Thus, *S. stutzeri* strains NRCB010 and NRCB025 are promising for agronomic application.

Soil texture, SOM, available N, and pH affect the ability of PGPR to mitigate N₂O emissions (11–13, 15). The differences in soil texture, SOM, and available N between the Yixing and Nanjing soils affected the ability of strains NRCB010 and NRCB025 to reduce N₂O to N₂. The Yixing soil was fine-textured, and the Nanjing soil was coarse-textured. This result indicates that the Yixing soil might restrain gas diffusivity and enhance N₂O reduction to N_2_ through complete denitrification relative to the Nanjing soil. The SOM in the Yixing soil was significantly higher than that in the Nanjing soil (Table 3), and the contents of NO_3_^–^-N and NO_2_^–^-N in the Yixing soil were significantly lower than those in the Nanjing soil (Table 3); hence, the cumulative N₂O emissions were much greater from the Nanjing soil than from the Yixing soil. NRCB010 and NRCB025 contained the *nosZ* gene (Fig. S4) but showed different reduction rates in N₂O emissions from the same soil (Fig. 2). This result indicates that *nosZ* expression levels or N₂O reductase activity may differ between NRCB010 and NRCB025 (4, 20). In addition, tomato biomass in the Yixing soil was higher than that in the Nanjing soil (Fig. 1). This result indicates that the tomato plants may have taken up more nitrogen from the Yixing soil than the Nanjing soil, further decreasing the Yixing soil’s available N. Consistent with findings in the literature (2), NRCB010 and NRCB025 changed soil pH, SOM, NH_4_^+^, and NO_3_^–^ contents (Table 1), indicating that PGPR may change the effective substrate for nitrification- and denitrification-related microorganisms, affecting N₂O emissions (11–13). Therefore, inoculation with NRCB010 decreased the cumulative N₂O emissions from the Yixing and Nanjing soils. Inoculation with NRCB025 decreased N₂O emissions from the Nanjing soil only (Fig. 2), and N₂O flux and cumulative emissions were much greater in the Nanjing soil than in the Yixing soil (Fig. 2). These results suggest that NRCB010 and NRCB025 possess N₂O mitigation abilities, but their reduction capabilities are strain-specific and soil texture-dependent.

**TABLE 3:** Physicochemical properties of soils for greenhouse experiments^*a*^

Soil source	Soil texture	Bulk density	pH	SOM	NH_4_^+^-N	NO_3_^-^-N	NO_2_^-^-N	Available *P*	Exchangeable K	Exchangeable Ca	Exchangeable mg
		(g cm^−3^)	(H_2_O)	(g kg^−1^)	(mg kg^−1^)	(mg kg^−1^)	(mg kg^−1^)	(mg kg^−1^)	(mg kg^−1^)	(mg kg^−1^)	(mg kg^−1^)
Yixing	Fine	0.85	7.08a	12.83a	7.00a	16.22b	1.47b	41.49b	256.70b	1105.2a	1033.4b
Nanjing	Coarse	1.13	6.81a	11.07b	7.41a	17.59a	4.82a	68.15a	280.40a	1090.2b	1054.2a

^*a*^ Values are mean ± SE (*n* = 4). Letters after the data of the same index at the same soil denote significant differences between different treatments, revealed by a *t* test (*P* < 0.05). NH_4_^+^-N: ammonium nitrogen; NO_3_^-^-N: nitrate nitrogen; NO_2_^-^-N: nitrite nitrogen.

### Effects of PGPR on soil microbial community and N-cycle functional genes

PGPR alters bacterial community composition and diversity, which may be closely related to N₂O generation and consumption. Bacterial community composition and diversity differed between the Yixing and Nanjing soils, and inoculation with NRCB025 and NRCB010 also changed the bacterial community diversity (Fig. 3 and 4). The relative abundance of Proteobacteria and Actinobacteria generally increased after inoculation with the two *S*. *stutzeri* strains (Fig. 4). Most denitrifying bacteria belong to Proteobacteria, Actinobacteria, Firmicutes, and Cyanobacteria, and an increased abundance of these phyla may be conducive to enhanced denitrification (25). The relative abundances of certain genera belonging to Proteobacteria, such as *Sphingomonas*, *Pseudomonas*, and *Rhodanobacter*, contained N₂O-producing and consuming species (26). The abundance of denitrification functional genes increased to varying degrees (NO_3_^–^ reduction, NO_2_^–^ reduction, and N₂O reduction) in both soils after inoculation with NRCB010 and NRCB025 (Fig. 5), improving N₂O mitigation (10).

NH_3_ oxidation is the rate-limiting step in nitrification and is important for producing nitrification-related N₂O (27). PGPR changes N-cycle functional gene copy numbers and relative quantities (1, 28). The abundance of N-cycle function genes directly regulates the production and consumption of N₂O (1, 28). Inoculation with NRCB010 significantly decreased bacterial *amoA* gene copy numbers in both soils, and NRCB025 significantly decreased archaeal and bacterial *amoA* gene copy numbers in the Nanjing soil at 15–18 DAI (Fig. S2B and C, S3B and C). Inoculation with NRCB010 significantly decreased *amoB* gene copy numbers in the Yixing soil at 31 DAI (Fig. S2D). Furthermore, the (*amoA + amoB*)/(*nosZ*I + *nosZ*II) ratios decreased after inoculation with NRCB010 (Fig. 6A and C); thus, NRCB010 might have changed the denitrification that contributes to N₂O. These results indicate that inoculation with NRCB010 and NRCB025 may inhibit N₂O production via nitrification (1, 28). Inoculation with NRCB010 significantly increased the n*osZ*II gene copy number in the Yixing soil (Fig. S2H), and inoculation with NRCB010 and NRCB025 significantly increased the *nosZ*I and *nosZ*II gene copy numbers, respectively, in the Nanjing soil (Fig. S3G and H). The (*nirS + nirK*)/(*nosZ*I + *nosZ*II) ratios decreased after inoculation with NRCB010 and NRCB025 (Fig. 6B and D). A lower *nir*/*nosZ* ratio indicates more N₂O consumption (2), decreasing N₂O emissions from soil. These results indicate that both strains may promote N₂O consumption via denitrification, decreasing N₂O emissions by converting additional N₂O into N_2_ (10, 29). Therefore, inoculation with *S. stutzeri* strains decreased N₂O from two vegetable agricultural soils with contrasting textures, possibly through decreasing N₂O production and increasing N₂O consumption.

### Key factors governing PGPR to mitigate N₂O emissions from vegetable agricultural soils

In this study, the inoculation with PGPR strains decreased N₂O emissions from soils. Abiotic (e.g., soil pH and SOM) and biological factors (e.g., bacterial community composition, diversity, and N-cycle gene copy numbers) influenced soil N₂O emissions. The SEM revealed that distinct factors regulated N₂O emissions from these two vegetable agricultural soils (Fig. 7). In the control treatment, N₂O emissions were positively and directly affected by *nosZ*II in the Yixing soil (Fig. 7A) but were not influenced by *nosZ*II in the Nanjing soil (Fig. 7G). This result indicates that the Yixing soil may have a greater ability than the Nanjing soil to reduce N₂O to N_2_, owing to the greater contribution of *nosZ*II than that in the Nanjing soil (10, 29), verified by the N₂O emissions from the Yixing soil being much lower than those from the Nanjing soil (Fig. 2). These results further indicate that the effect of PGPR on N₂O mitigation is soil texture-dependent.

SEM further revealed a clear distinction in the key variables explaining N₂O emissions among the control, NRCB010, and NRCB025 treatments (Fig. 7). In both soils, N₂O emissions were positively and directly influenced by *amoA* gene copy numbers and were negatively and directly influenced by *nosZ* (I or II) after inoculation with NRCB010 (Fig. 7C and I). This result indicates that NRCB010 decreased soil N₂O emission mainly by inhibiting nitrification and stimulating N₂O reduction to N₂ (2, 10). The decline in the (*amoA + amoB*)/(*nosZ*I + *nosZ*II) ratio further confirmed this finding (Fig. 6A and C). Moreover, N₂O emissions were positively directly influenced by *nirK* gene copy numbers after inoculation with NRCB025 (Fig. 7E and K). Approximately one-third of *nirS/K*-denitrifying microorganisms do not have the *nosZ* gene (27, 30), implying that NRCB025 decreases soil N₂O emissions mainly by inhibiting N₂O generation in the denitrification. The (*nirS + nirK*)/(*nosZ*I + *nosZ*II) ratio decreased further, confirming this assumption (Fig. 6D). These results demonstrate that the effects of PGPR on N₂O mitigation are strain-specific in this study. Moreover, our results showed that the low N₂O emissions from soils inoculated with NRCB010 and NRCB025 were mainly attributed to the decreased (*amoA + amoB*)/(*nosZ*I + *nosZ*II) and (*nirS + nirK*)/(*nosZ*I + *nosZ*II) ratios and the key soil physicochemical properties.

Overall, inoculation with *S*. *stutzeri* strains enhanced vegetable crop productivity and decreased N₂O emissions from two vegetable agricultural soils with contrasting textures. Moreover, the N₂O-mitigating effect varied depending on soil textures and the individual strain after inoculation. The N₂O-mitigating effect was achieved possibly by altering the soil microbial community composition and gene abundance involved in nitrification and denitrification after inoculation. However, further research is necessary to determine whether their beneficial effects are maintained in other vegetable agricultural soils and for other textures and how to combine efficient strains with different carriers (e.g., organic manure, biogas residue digestate, and biochar). Further research should also investigate how to sustain or enhance these beneficial effects at the field scale.

## MATERIALS AND METHODS

### Candidate strains

*S. stutzeri* strains NRCB010 and NRCB025 isolated from rice rhizosphere soil collected in Yixing, Jiangsu Province, China (31^°^12′ N, 119^°^52′ E) (21) were used as candidate strains.

### Soil sampling

Vegetable soil from Yixing (N 31^°^12′, E 119^°^52′; fine-textured soil) and Nanjing (N 32^°^4′, E 118^°^38′; coarse-textured soil), Jiangsu, China, were selected for greenhouse experiments. This study collected 0–20 cm topsoil, air-dried, sieved through a 2 mm sieve, then maintained at 25 ± 2°C for further use. The soil physicochemical properties are listed in Table 3.

### Greenhouse pot experiment

The pot experiments were performed in two batches in a greenhouse at Nanjing Tech University, Nanjing, China (32^°^4′ N, E 118^°^38′). Organic fertilizer (STANLEY, N + P_2_O_5_ + K_2_O 5% and organic matter 45%) and compound fertilizer (Fulaishun, N-P_2_O_5_-K_2_O = 15:15:15) were applied to each soil: 0.4 g kg^–1^ air-dried soil and 6 g kg^–1^ air-dried soil, respectively. The fertilizer was mixed thoroughly with soil and placed in plastic pots (11.5 cm top diameter × 14 cm depth), using 445 and 460 g air-dried soil per pot for the Yixing and Nanjing soils, respectively.

Tomato seeds (*Solanum lycopersicum* L. cv. Zhongshu No. 4) were purchased from Fanyu Seed Co., Ltd. (Tianjin, China). Seed preparation, strain suspension, and inoculation procedures were slightly modified from previously described methods (5, 21). The NRCB010 and NRCB025 strains were cultured in Nutrient broth with sodium nitrate and sodium succinate (NBNS) liquid medium (21), centrifuged, resuspended in sterile one-tenth Hoagland solution (Hope Biotechnology Co. Ltd. Beijing) to an OD600 value approximately equal to 1.0 (about Log 9 colony forming units per mL), and diluted 10 times for subsequent use. The seeds were surface-sterilized in a 2.5% sodium hypochlorite solution for 10 min, washed with sterile distilled water, and germinated (25°C ± 2°C) in sterile Petri dishes for 3 d. The uniform germinated seeds were broadcast into the soil, and 10 mL 10-times-diluted strain suspension was inoculated into the soil. The seeds and soil of the control were treated with Hoagland solution without a strain. Each treatment consisted of four pots/replicates, and each pot had three tomato seedlings. One of three plants and corresponding rhizosphere soil were sampled each time, yielding 12 samples per treatment in total. The greenhouse was temperature-controlled at 25°C ± 2°C and maintained under a 14/10 h (light/dark) cycle.

### Plant and soil sampling and N₂O flux measurement in greenhouse pot experiments

Tomato seedlings and soil were sampled at 18, 25, and 31 DAI and 15, 22, and 29 DAI in the Yixing and Nanjing soils, respectively. Tomato plant height, stem diameter, leaf length, and dry weight were measured. Soil pH, soil organic matter, ammonium N (NH_4_^+^-N), and nitrate N (NO_3_^–^-N) were determined (31).

N₂O flux was examined at 0, 2, 4, 6, 8, 10, 13, 16, 19, 23, 27, and 31 DAI and at 0, 2, 4, 6, 8, 10, 12, 15, 18, 22, and 29 DAI in the Yixing and Nanjing soils, respectively. Each pot was placed in a sealed chamber (15.5 × 15.5 × 25.7 cm); a gas sample was collected at 0, 15, and 30 min; and the samples were measured using a gas chromatograph with an electron capture detector (Agilent 7890B, USA) (32). N₂O flux and cumulative emissions were calculated as described previously (32). Each treatment consisted of four replicates (pots).

### 16S rRNA gene sequencing and real-time quantitative PCR

Tomato rhizosphere soil was collected at 18, 25, and 31 DAI and at 15, 22, and 29 DAI in the Yixing and Nanjing soils, respectively. The collected soil samples were stored at −80°C until DNA extraction. According to the manufacturer’s protocols, total genomic DNA was extracted from 0.5 g of each soil sample by using the FastDNA Spin Kit for Soil (MP Biomedicals Co., Ltd., Santa Ana, CA, USA). The 16S rRNA gene was used for high-throughput sequencing and real-time quantitative PCR (qPCR) analysis. DNA samples were qualified and sequenced by Guangzhou Gene Denovo Biotechnology Co., Ltd (Guangzhou, China). The V3−V4 regions of the 16S rRNA gene were amplified using PCR, with amplification yielding approximately 466 bp products. Purified PCR products were obtained using the Illumina NovaSeq platform. This study used QIIME2 (V1.9.1, http://qiime.org/scripts/split/libraries/fastq.html) and FLASH (VI. 2.7, http://ccb.jhu.edu/software/FLASH/) for the original quality control of sequence data, removal of chimeras, and sequence denoising to generate amplified sequence variants. Species annotation information was added to sequence variants by using the SILVA database (V138.1, https://www.arb-silva.de/) (33).

The copy numbers of the *16S rRNA*, *amoA*, *amoB*, *nirS*, *nirK*, and *nosZ* genes in soil samples were measured using a CFX96 Touch Real-time PCR Detection System (Bio-Rad, Hercules, CA, USA). The DNA samples were diluted to 10 ng µL^–1^ with distilled sterile H_2_O. Standard curves were generated using 10-fold dilutions of plasmid DNA. The amplification efficiency ranged from 84.1% to 99.5%, and the *R*^2^ value ranged from 0.997 to 0.999. Each treatment was quantified using four biological and two technical replicates. Target gene copy numbers were calculated from standard curves and presented per gram of dry-weight soil (copies g^–1^ dw soil) (34). The primer sequences, reaction system, and amplification conditions used for qPCR are shown in Tables S2 and S3.

### Meta 16S rRNA sequencing analysis

Raw sequencing data were denoised using the UPARSE standard operating procedures. High-throughput sequencing data were analyzed using VSEARCH (version vsearch-2.21) (35). Using the fastq merge pairs command to combine two-ended sequence files, double-ended primers and barcodes were removed, and the quality control reference value was set at <10. The UCHIME method removed chimeras and assigned an operational taxon based on 97% similarity (i.e., OTU). The classification status of the OTUs was annotated using SINTAX.

### Structural equation modeling

SEM was used to evaluate relationships among soil physicochemical properties (pH and SOM), N-cycle functional genes, and N₂O emissions in response to the different treatments in the Yixing and Nanjing soils. The analysis used Amos Graphics (version 24.0; Amos Development Corporation, Meadville, PA, USA).

### Statistical analysis

Pearson’s correlation analysis, one-way ANOVA, independent samples *t* test (*P* < 0.05), and Duncan’s multiple range test (*P* < 0.05) were performed using SPSS version 26.0. Alpha diversity (Shannon, Chao1, and Simpson indices) and β diversity (Bray-Curtis) were calculated using R software (version 3.5.0; https://www.r-project.org/). The Venn diagrams were constructed of unique and common elements using the VennDiagram package. The relative abundance of species was presented as the specific community composition of the bacteria by using ggplot2. Principal coordinate analysis was used to calculate the Bray-Curtis distances by using the Vegan package. Heatmaps of the top 20 species with relative abundance ratios at the genus level were constructed using the heatmap package. Based on the annotation results of the *16S rRNA* sequences, a prediction of the function of the microbial community was performed using the Functional Annotation of Prokaryotic Taxa and visualized using a heatmap.

## Data Availability

Raw data for N₂O measurements and tomato growth indicators are available upon reasonable request. Meta 16S rRNA sequences have been deposited in the National Center of Biotechnology Information database (Accession number: PRJNA1006208).

## SUPPLEMENTAL MATERIAL

Supplemental material spectrum.00186-24-s0001.docx Fig. S1 to S4; Tables S1 to S3.

ASM does not own the copyrights to Supplemental Material that may be linked to, or accessed through, an article. The authors have granted ASM a non-exclusive, world-wide license to publish the Supplemental Material files. Please contact the corresponding author directly for reuse.
