# Supplementary material for: Inoculation with Stutzerimonas stutzeri strains decreases N₂O emissions from vegetable soil by altering microbial community composition and diversity
Source: Microbiol Spectr. 2024 Mar 21;12(5):e00186-24. doi: 10.1128/spectrum.00186-24 (PMC11064591; doi:10.1128/spectrum.00186-24)
Supplement: Supplemental material — Fig. S1 to S4; Tables S1 to S3. [file spectrum.00186-24-s0001.docx]

**Supplementary figures**


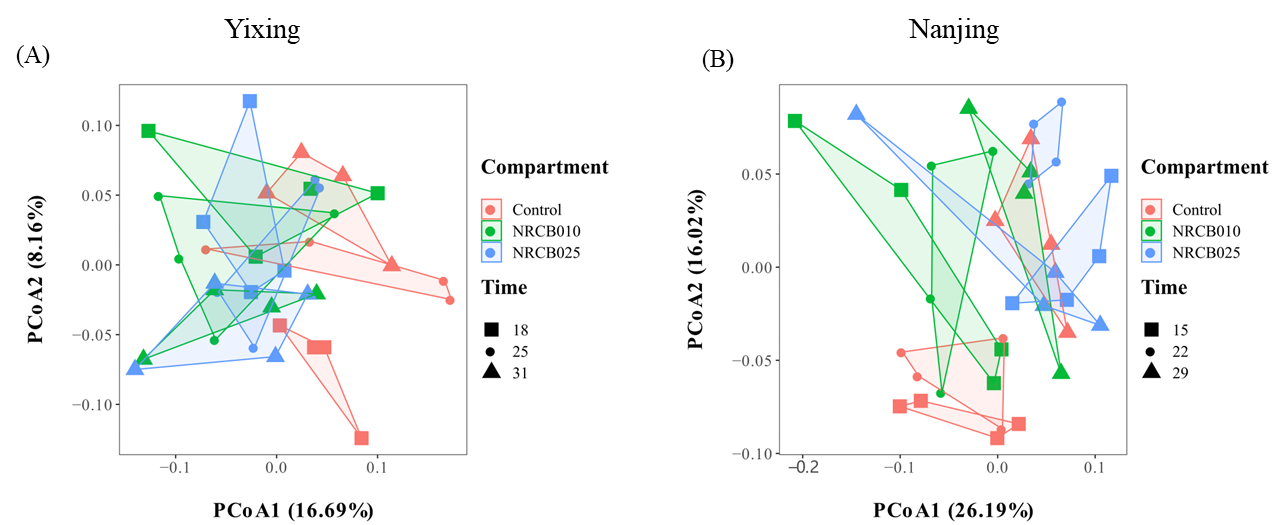


**Fig. S1** Principal component analysis (PCoA) of soil bacterial community diversity based on weighted UniFrac distances under different treatments


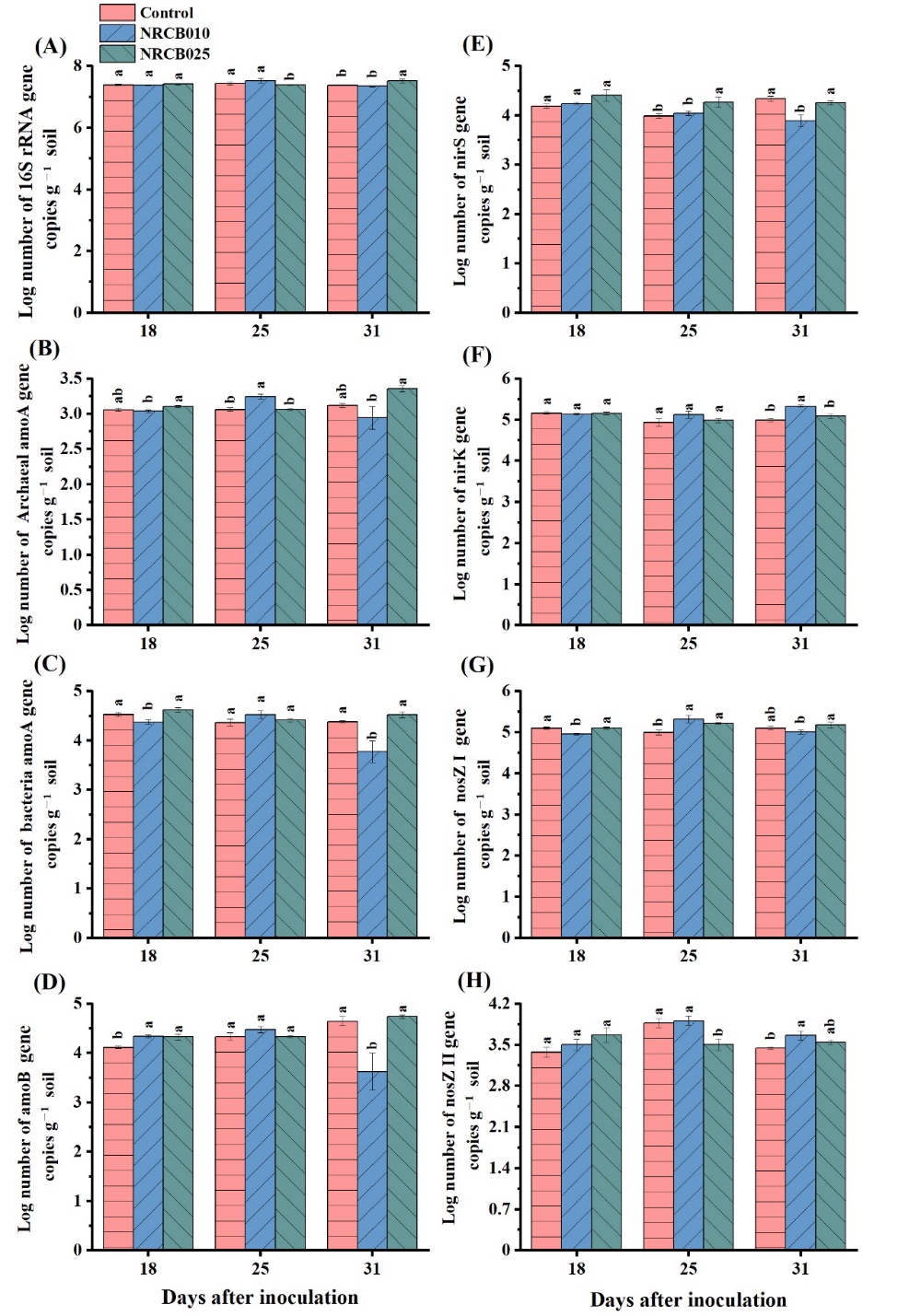


**Fig. S2** Dynamics of the copy number of *AOA*, *AOB*, *amoB*, *nirK*, *nirS*, *nosZ*I, and *nosZ*II under different treatments in Yixing soil. Values are means ± standard error (n = 4). Letters above the bars at the same time denote significant differences between treatments revealed by Duncan's post hoc test (*P* < 0.05).


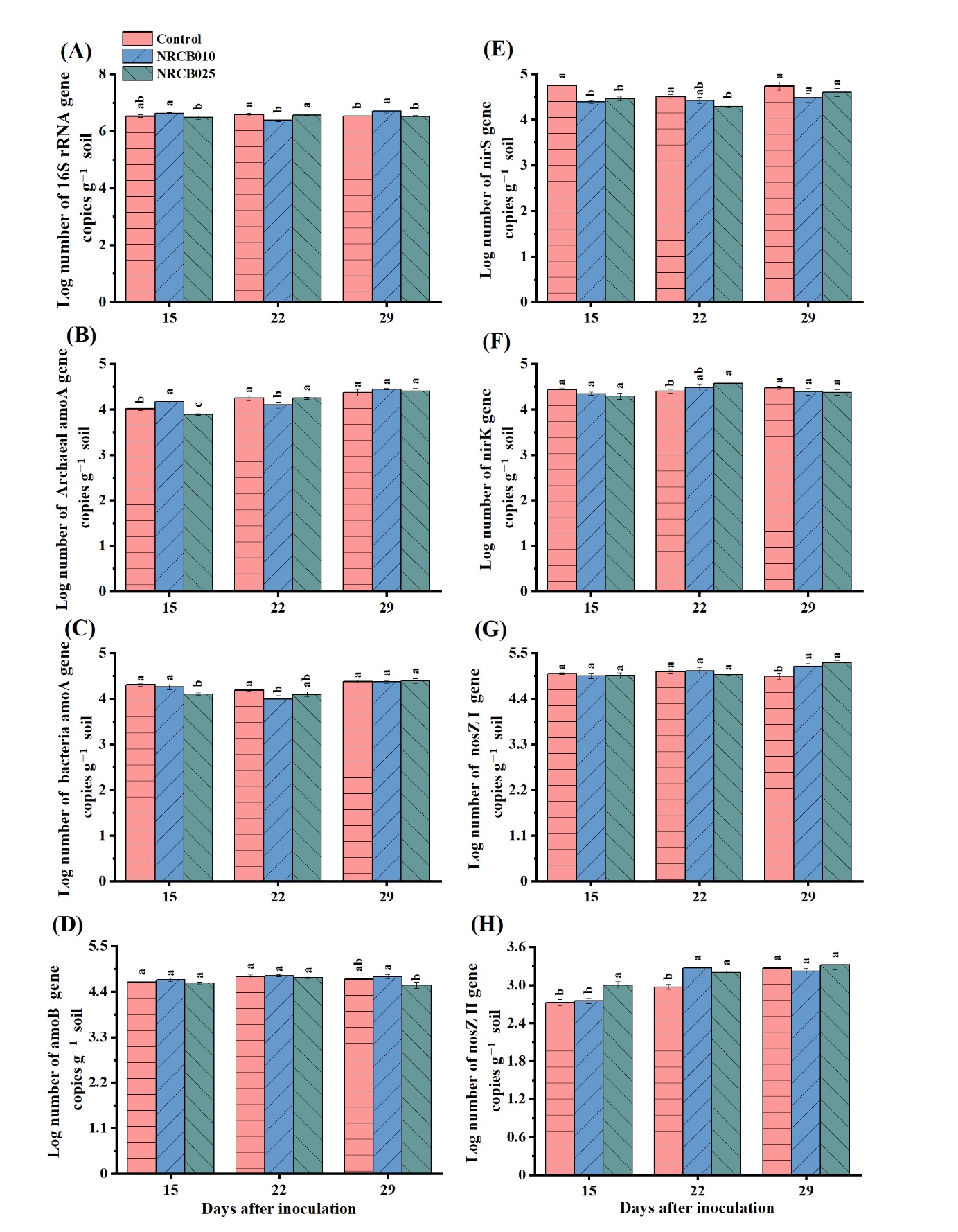


**Fig. S3** Dynamics of the copy number of *AOA*, *AOB*, *amoB*, *nirK*, *nirS*, *nosZ*I, and *nosZ*II under different treatments in Nanjing soil. Values are means ± standard error (n = 4). Different letters above the bars at the same time denote significant differences between treatments by Duncan's post-hoc test (*P* < 0.05).

**
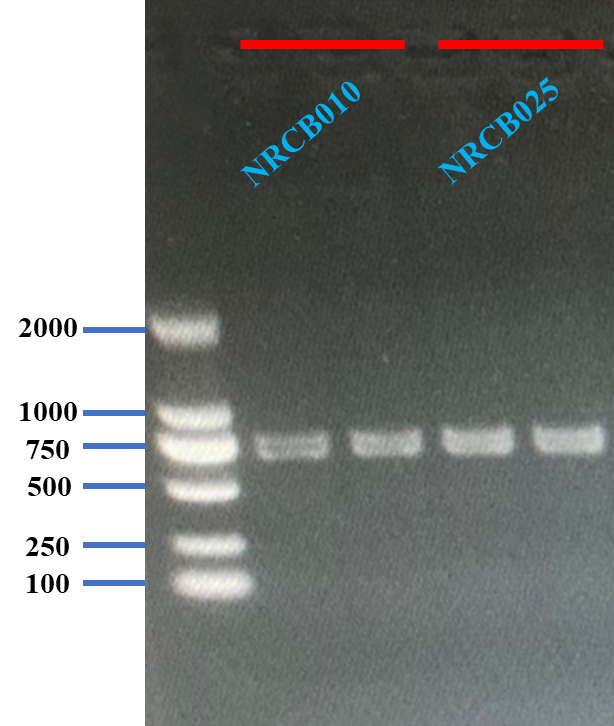
**

**Fig. S4** Nitrous oxide reduction gene (*nosZ*) amplification of *Stutzerimonas stutzeri* NRCB010 and NRCB025.

**Supplementary tables**

**Table S1** Physicochemical properties of tomato soil after inoculation with *Stutzerimonas stutzeri*

| Treatment |  | NH_4_^+^-N  (mg kg^-1^) |  |  | NO_3_^-^-N  (mg kg^-1^) |  |  |
| --- | --- | --- | --- | --- | --- | --- | --- |
| Yixing Soil |  |  |  |  |  |  |  |
|  | DAI | 18 | 25 | 31 | 18 | 25 | 31 |
| Control |  | 8.9±0.0a | 9.0±0.1a | 7.0±0.1a | 16.6±1.3a | 17.3±0.8a | 17.1±0.4a |
| NRCB010 |  | 9.2±0.3a | 9.2±0.2a | 7.2±0.1a | 17.6±0.6a | 16.7±1.0a | 16.6±2.6a |
| NRCB025 |  | 8.8±0.1a | 9.1±0.2a | 6.9±0.1a | 18.5±0.8a | 16.5±1.1a | 16.9±3.7a |
| Nanjing Soil |  |  |  |  |  |  |  |
|  | DAI | 15 | 22 | 29 | 15 | 22 | 29 |
| Control |  | 8.7±0.1a | 6.7±0.2b | 8.6±0.1a | 14.8±1.6a | 16.2±2.2a | 19.0±6.2a |
| NRCB010 |  | 8.7±0.1a | 8.6±0.0a | 8.2±1.0a | 19.9±1.5a | 15.0±1.0a | 13.6±4.4a |
| NRCB025 |  | 8.9±0.2a | 8.6±0.1a | 7.5±0.5a | 18.6±1.8a | 14.8±2.6a | 15.3±2.6a |

Values are means ± standard error (n = 4). Letters after the data in each volume denote significant differences between different treatments revealed by Duncan's post hoc test (*P* < 0.05). DAI: days after inoculation; NH_4_^+^-N: ammonium nitrogen; NO_3_^-^-N: nitrate nitrogen.

**Table S2** Bacterial strains and primers used for the construction of qPCR-standards.

| Gene | Primer | Sequence | Amplicon size (bp) | Strain | Cloning vector ^a^ | Reference |
| --- | --- | --- | --- | --- | --- | --- |
| *16S rRNA* | 341F  806R | CCTACGGGNGGCWGCAG GGACTACHVGGGTATCTAAT | 466 | DH5α | pMD19^®^ | (1) |
| *Archaeal* *amoA* | Arch-amoAF  Arch-amoAR | STAATGGTCTGGCTTAGACG GCGGCCATCCATCTGTATGT | 635 | DH5α | pMD19^®^ | (2) |
| *Bacterial amoA* | amoA-1F  amoA-2R | GGGGTTTCTACTGGTGGT CCCCTCKGSAAAGCCTTCTTC | 491 | DH5α | pMD19^®^ | (3) |
| *amoB* | cmx_amoB-148F cmx_amoB_485R | TGGTAYGAYACNGAATGGG CCCGTGATRTCCATCCA | 337 | DH5α | pMD19^®^ | (4) |
| *nirS* | nirS-cd3AF  nirS-R3cd | GTSAACGTSAAGGARACSGG GASTTCGGRTGSGTCTTGA | 500 | DH5α | pMD19^®^ | (5) |
| *nirK* | nirK -F1aCu  nirK -R3Cu | ATCATGGTSCTGCCGCG  GCCTCGATCAGRTTGTGGTT | 473 | DH5α | pMD19^®^ | (6) |
| *nosZ*I | nosZ2F  nosZ2R | CGCRACGGCAASAAGGTSMSSGT  CAKRTGCAKSGCRTGGCAGAA | 267 | DH5α | pMD19^®^ | (7) |
| *nosZ*II | nosZ912F  nosZ1853R | CGTCCCCGGCCTCGTGTA  GAGCAGAAGTTCGTGCAGTAGTAGGG | 880 | DH5α | pMD19^®^ | (8) |

**Table S3** Quantitative PCR reaction mixtures and thermal profiles for the different target genes.

| Target gene | Reaction mixture | Volumes (25 µl) | Thermal profile |
| --- | --- | --- | --- |
| *16S rRNA* | TB Green® Premix Ex Taq™ II (Tli RNaseH Plus)  341f (10 µM)  806R (10 µM)  ddH_2_O  template (10 ng µL^-1^) | 12.5  1  1  5.5  5 | 95 °C 1 min  95 °C 5 s  55°C 30 s  72°C 45 s  Go to 2, 40 cycles  95 °C 10 s  Melt cure 65.0 to 95.0 °C increment 0.5 °C for 0.05 s, end |
| *Archaeal amoA* | TB Green® Premix Ex Taq™ II (Tli RNaseH Plus)  Arch-amoAF (10 µM)  Arch-amoAR (10 µM)  ddH_2_O  template (10 ng µL^-1^) | 12.5  1  1  5.5  5 | 95 °C 1 min  95 °C 5 s  55 °C 30 s  72 °C 45 s  Go to 2, 40 cycles  95 °C 10 s  Melt cure 65.0 to 95.0 °C increment 0.5 °C for 0.05 s, end |
| *Bacterial amoA* | TB Green® Premix Ex Taq™ II (Tli RNaseH Plus)  amoA-1F (10 µM)  amoA-2R (10 µM)  ddH_2_O  template (10 ng µL^-1^) | 12.5  1  1  5.5  5 | 95 °C 1 min  95 °C 5 s  55 °C 30 s  72 °C 45 s  Go to 2, 40 cycles  95 °C 10 s  Melt cure 65.0 to 95.0 °C increment 0.5 °C for 0.05 s, end |
| *amoB* | TB Green® Premix Ex Taq™ II (Tli RNaseH Plus)  cmx_amoB-148F (10 µM)  cmx_amoB_485R (10 µM)  ddH_2_O  template (10 ng µL^-1^) | 12.5  1  1  5.5  5 | 95 °C 1 min  95 °C 5 s  55 °C 30 s  72 °C 30 s  Go to 2, 40 cycles  95 °C 10 s  Melt cure 65.0 to 95.0 °C increment 0.5 °C for 0.05 s, end |
| *nirS* | TB Green® Premix Ex Taq™ II (Tli RNaseH Plus)  nirS-cd3AF (10 µM)  nirS-R3cd (10 µM)  ddH_2_O  template (10 ng µL^-1^) | 12.5  1  1  5.5  5  2 | 95 °C 1 min  95 °C 5 s  55°C 30 s  72°C 45 s  Go to 2, 40 cycles  95 °C 10 s  Melt cure 65.0 to 95.0 °C increment 0.5 °C for 0.05 s, end |
| *nirK* | TB Green® Premix Ex Taq™ II (Tli RNaseH Plus)  nirK -F1aCu (10 µM)  nirK -R3aCu (10 µM)  ddH_2_O  template (10 ng µL^-1^) | 12.5  1  1  5.5  5 | 95 °C 1 min  95 °C 5 s  55 °C 30 s  72 °C 60 s  Go to 2, 40 cycles  95 °C 10 s  Melt cure 65.0 to 95.0 °C increment 0.5 °C for 0.05 s, end |
| *nosZ*I | TB Green® Premix Ex Taq™ II (Tli RNaseH Plus)  nosZ2F (10 µM)  nosZ2R (10 µM)  ddH_2_O  Template (10 ng µL^-1^) | 12.5  1  1  5.5  5 | 95 °C 1 min  95 °C 5 s  55 °C 30 s  72 °C 60 s  Go to 2, 40 cycles  95 °C 10 s  Melt cure 65.0 to 95.0 °C increment 0.5 °C for 0.05 s, end |
| *nosZ*II | TB Green® Premix Ex Taq™ II (Tli RNaseH Plus)  nosZ912F (10 µM)  nosZ1853R (10 µM)  ddH_2_O  Template (10 ng µL^-1^) | 12.5  1  1  5.5  5 | 95 °C 1 min  95 °C 5 s  55 °C 30s  72 °C 1:20 s  Go to 2, 40 cycles  95 °C 10 s  Melt cure 65.0 to 95.0 °C increment 0.5 °C for 0.05 s, end |

Amplifications of these genes were performed in duplicates under the above cyclic conditions.**Supplementary references**

1. Nadkarni MA, Martin FE, Jacques NA, Hunter N. 2002. Determination of bacterial load by real-time PCR using a broad-range (universal) probe and primers set. Microbiology-Sgm 148:257-266.

2. Francis CA, Roberts KJ, Beman JM, Santoro AE, Oakley BB. 2005. Ubiquity and diversity of ammonia-oxidizing archaea in water columns and sediments of the ocean. Proc Natl Acad Sci U S A 102:14683-8.

3. Rotthauwe JH, Witzel KP, Liesack W. 1997. The ammonia monooxygenase structural gene amoA as a functional marker: Molecular fine-scale analysis of natural ammonia-oxidizing populations. Applied and Environmental Microbiology 63:4704-4712.

4. Cotto I, Dai Z, Huo L, Anderson CL, Vilardi KJ, Ijaz U, Khunjar W, Wilson C, De Clippeleir H, Gilmore K, Bailey E, Pinto AJ. 2020. Long solids retention times and attached growth phase favor prevalence of comammox bacteria in nitrogen removal systems. Water Research 169:115268.

5. Throback IN, Enwall K, Jarvis A, Hallin S. 2004. Reassessing PCR primers targeting nirS, nirK and nosZ genes for community surveys of denitrifying bacteria with DGGE. Fems Microbiology Ecology 49:401-417.

6. Hallin S, Lindgren PE. 1999. PCR detection of genes encoding nitrile reductase in denitrifying bacteria. Applied and Environmental Microbiology 65:1652-1657.

7. Henry S, Bru D, Stres B, Hallet S, Philippot L. 2006. Quantitative detection of the nosZ gene, encoding nitrous oxide reductase, and comparison of the abundances of 16S rRNA, narG, nirK, and nosZ genes in soils. Applied and Environmental Microbiology 72:5181-5189.

8. Sanford RA, Wagner DD, Wu QZ, Chee-Sanford JC, Thomas SH, Cruz-Garcia C, Rodriguez G, Massol-Deya A, Krishnani KK, Ritalahti KM, Nissen S, Konstantinidis KT, Loffler FE. 2012. Unexpected nondenitrifier nitrous oxide reductase gene diversity and abundance in soils. Proceedings of the National Academy of Sciences of the United States of America 109:19709-19714.
